# Vitamin D Status during Pregnancy and the Risk of Subsequent Postpartum Depression: A Case-Control Study

**DOI:** 10.1371/journal.pone.0080686

**Published:** 2013-11-27

**Authors:** Nina O. Nielsen, Marin Strøm, Heather A. Boyd, Elisabeth W. Andersen, Jan Wohlfahrt, Marika Lundqvist, Arieh Cohen, David M. Hougaard, Mads Melbye

**Affiliations:** 1 Department of Epidemiology Research, Statens Serum Institut, Copenhagen, Denmark; 2 National Institute of Public Health, University of Southern Denmark, Copenhagen, Denmark; 3 Department of Clinical Biochemistry and Immunology, Statens Serum Institut, Copenhagen, Denmark; UCL Institute of Child Health, University College London, United Kingdom

## Abstract

Epidemiological studies have provided evidence of an association between vitamin D insufficiency and depression and other mood disorders, and a role for vitamin D in various brain functions has been suggested. We hypothesized that low vitamin D status during pregnancy might increase the risk of postpartum depression (PPD). The objective of the study was thus to determine whether low vitamin D status during pregnancy was associated with postpartum depression. In a case-control study nested in the Danish National Birth Cohort, we measured late pregnancy serum concentrations of 25[OH]D3 in 605 women with PPD and 875 controls. Odds ratios [OR) for PPD were calculated for six levels of 25[OH]D3. Overall, we found no association between vitamin D concentrations and risk of PPD (p = 0.08). Compared with women with vitamin D concentrations between 50 and 79 nmol/L, the adjusted odds ratios for PPD were 1.35 (95% CI: 0.64; 2.85), 0.83 (CI: 0.50; 1.39) and 1.13 (CI: 0.84; 1.51) among women with vitamin D concentrations < 15 nmol/L, 15–24 nmol/L and 25–49 nmol/L, respectively, and 1.53 (CI: 1.04; 2.26) and 1.89 (CI: 1.06; 3.37) among women with vitamin D concentrations of 80–99 nmol/L and ≥ 100 nmol/L, respectively. In an additional analysis among women with sufficient vitamin D (≥ 50 nmol/L), we observed a significant positive association between vitamin D concentrations and PPD. Our results did not support an association between low maternal vitamin D concentrations during pregnancy and risk of PPD. Instead, an increased risk of PPD was found among women with the highest vitamin D concentrations.

## Introduction

Postpartum depression (PPD) is a significant public health problem estimated to affect 10–15% of women worldwide [1]. In fact, a recent systematic review carried out among women from rural areas reported PPD prevalences as high as 31.3% and 21.5% in developing and developed countries, respectively [2]. PPD is defined as a nonpsychotic depressive episode that starts in the postpartum period, generally defined as the year after delivery [1], [2]. The disorder is characterized by emotional lability, tearfulness, loss of appetite, sleep disturbance, poor concentration and memory, fatigue, irritability, sense of guilt, and feelings of inadequacy and inability to take care of the infant [2]. PPD can have devastating effects on mother-infant interactions during the first year of life [3] and can influence the child’s cognitive and emotional development [4]. Among the most commonly reported risk factors associated with PPD are a past history of depression or other psychiatric illness, depression during pregnancy, lack of social support, recent life stresses, child care stress, difficult infant temperament and fatigue [1], [2], [4], [5].

The synthesis of vitamin D in the skin depends on ultraviolet B radiation from sunlight [6]. Ultraviolet B radiation acts on a cholesterol metabolite (7-dehydroxycholesterol) in the epidermis, which leads to the production of vitamin D3. Subsequent hydroxylation in the liver produces the inactive form of vitamin D, 25-hydroxyvitamin D3 (25(OH)D3), and further hydroxylation in the kidneys yields the biologically active form, 1,25-dihydroxyvitamin D3 (1,25(OH)2D3). Vitamin D concentration are thus associated with the duration of the photoperiod and influenced by season and latitude [7].

The profusion of vitamin D receptors and 1α-hydroxylase, the enzyme responsible for the formation of the active vitamin, in the human brain suggests that vitamin D may be important in certain mental processes [8]. Although the mechanism whereby low vitamin D concentrations might produce depression has yet to be demonstrated, a lack of stimulation of vitamin D receptors as a consequence of vitamin D deficiency could result in improper functioning of hormonal processes in the brain that prevent mood disorders [9], making such a link biologically plausible. Associations between low vitamin D levels, depression and other mood disorders are well-documented, with studies providing consistent and convincing evidence of an association, at least in the elderly and in populations with certain chronic diseases [10]–[14], despite differences in study design, approach to assessing mood/depressive symptoms, definition of low vitamin D levels, and choice of confounders.

However, studies of the effect of vitamin D supplementation on mood and depression have provided inconclusive results. In two studies of non-depressed subjects, supplementation enhanced mood [15] and improved wellbeing [16]. Two studies of subjects with depressive symptoms at baseline also reported positive effects of vitamin D supplementation; both studies showed a reduction in depressive symptoms in persons receiving supplements, particularly in those with more severe depression [17], and serum vitamin D concentrations <100 nmol/L at baseline [18]. On the other hand, a recent randomised, controlled trial found no effect of vitamin D supplementation on mental well-being among older women [19].

Low vitamin D levels may be a neglected risk factor for PPD. A high prevalence (5–20% in light-skinned populations and 30–70% in dark-skinned or veiled populations) of maternal vitamin D inadequacy (vitamin D levels <25–50 nmol/L) during pregnancy and at delivery has been demonstrated in various populations living at different latitudes [20], probably due to the demands of the developing foetus. Given the acknowledged high prevalence of low vitamin D levels among pregnant women and the frequent occurrence of PPD, the public health impact of an association between vitamin D deficiency and PPD is potentially enormous. However, to our knowledge, only one small exploratory study has been carried out to elucidate the effect of vitamin D insufficiency on the occurrence of PPD [21]. This study found a significant relationship between low 25 (OH)D concentrations and high Edinburgh Postpartum Depression Scale scores (indicative of PPD). PPD has been shown to occur most frequently during winter [22], [23], which coincides with the season where low vitamin D concentrations are most prevalent [7].

We hypothesized that women low in vitamin D are prone to depression, and that events around childbirth (which contribute to “baby blues” in many women) provide the extra thrust that pushes already at-risk vitamin D-insufficient women into clinically apparent depression, resulting in PPD. To test whether low vitamin D concentrations during pregnancy are associated with increased risk of PPD, we performed a large case-control study nested within the Danish National Birth Cohort (DNBC).

## Methods

### Ethics statement

At recruitment, women in the DNBC gave informed written consent to use of their biological samples, interview information and medical information in future studies. The study was approved by the Scientific Ethics Committee for the Capital City Region (Copenhagen, Denmark).

### Study design and participants

Study participants were selected from the DNBC. The DNBC is a cohort of Danish women established to allow investigation of the impact of a wide range of prenatal exposures on peri- and postpartum outcomes in the mothers and on childhood, adolescent and early adult health of the offspring [24]. More than 91,000 pregnant women recruited during the period 1996–2002 gave birth to nearly 94,000 children between 1997 and 2003. The women were interviewed and venous blood was collected at approximately weeks 10–12 and 25 of pregnancy.

To be eligible for inclusion in this study, the DNBC participant had to have 1) been born in Denmark; 2) had a singleton pregnancy; 3) delivered a living child; and 4) had blood collected late in pregnancy (ca. week 25). Women with anti-depressant use registered in the Danish Register of Medicinal Product Statistics in the year before delivery and women registered in the Central Psychiatric Register with mental illnesses prior to their DNBC pregnancy were excluded from the study.

Case women (women with PPD) were women who filled prescriptions for anti-depressive medications (any ATC code beginning with N06A registered in the Register of Medicinal Product Statistics) within one year after delivery but were not hospitalized for PPD, i.e. PPD requiring pharmacologic treatment without being severe enough to warrant admission to a psychiatric hospital. Women eligible to be controls were women who neither filled prescriptions for anti-depressive medications in the year after delivery nor were hospitalized for PPD. Controls were selected at random from among the eligible women in such a way that the resulting pool of controls had the same distribution of maternal ages and delivery years as the case group. A total of 605 cases met the inclusion criteria and were frequency matched to 875 controls.

### Vitamin D measurements

DNBC blood samples were collected in connection with routine visits to the general practitioner and sent to Statens Serum Institut (Copenhagen, Denmark) for processing by regular mail at ambient temperature. Samples could take up to 48 hours to reach Statens Serum Institut, but most arrived within 28 hours. Serum was stored at –80°C for 9–15 years before being thawed and analyzed for 25(OH)D3 and 25(OH)D2 by liquid chromatography-tandem mass spectrometry (LC-MSMS) using the “MSMS vitamin D” kit from Perkin Elmer (Waltham, MA). Briefly, 30 uL of serum were deproteinized in microtiter plates using 120 uL acetonitrile containing ^2^H_3_-25-OH vitamin D2 and ^2^H_3_-25-OH vitamin D3 as internal standards. The supernatants were transferred to fresh plates and dried under a gentle flow of nitrogen. Subsequently, the samples were derivatized using 4-phenyl-1,2,4-triazoline-3,5-dione (PTAD) dissolved in acetonitrile. The derivatization reaction was quenched with quench solution and the samples were subjected to LC- MSMS analysis. The LC-MSMS system consisted of a CTC PAL autosampler (CTC Analytics, Zwingen, Switzerland), a Thermo surveyor LC pump and a Thermo TSQ Ultra triple quadrupole mass spectrometer (Thermo Scientific Waltham, MA). Separation was achieved using a Thermo Gold C18 column (50×2.1 mm, 3 um). The following transitions were used: 619.3/298.1 and 607.3/298.1 for 25-OH vitamin D2 and D3 respectively, 622.3/301.1 and 610.3/298.1 for D2 and D3 internal standards, respectively, and 625.3/298.1 and 613.3/298.1 for D2 and D3 calibration standards respectively. However, although the method measured both 25(OH)D3 and 25(OH)D2, concentrations of 25(OH)D2 were negligible, and only 25(OH)D3 was considered in the analyses.

### Potential confounders

Potential confounders were identified a priori and included the season and gestational week in which the maternal blood sample was taken, as well as maternal parity, smoking during pregnancy, socioeconomic status, pre-pregnancy BMI, physical activity during pregnancy, social support and multivitamin supplement use during pregnancy. Information on parity, smoking, socioeconomic status, BMI, physical activity, social support and multivitamin use was derived from data collected during the two DNBC antenatal interviews. The variables were categorized as shown in **Table** **1**.

**Table 1:** Odds ratio (OR) for PPD by covariates and distribution of covariates amongst cases and controls.

	Cases(N = 605)	Controls(N = 875)	OR (95%CI)	P*
**Delivery year**	N (%)	N (%)		
1998	65 (11)	82 (9)	1.19 (0.81; 1.74)	0.93
1999	103 (17)	151 (17)	1.02 (0.74; 1.41)	
2000	146 (24)	216 (25)	1.01 (0.76; 1.36)	
2001	133 (22)	189 (22)	1.06 (0.78; 1.42)	
2002–2003	158 (26)	237 (27)	1 (reference)	
**Maternal age**				
18–25	83 (14)	111 (13)	1.13 (0.79; 1.62)	0.96
26–28	146 (24)	218 (25)	1.01 (0.74; 1.37)	
29–30	102 (17)	150 (17)	1.03 (0.73; 1.43)	
31–33	144 (24)	200 (23)	1.09 (0.80; 1.48)	
34+	130 (22)	196 (22)	1 (reference)	
**Pre-pregnancy BMI**				
Underweight	26 (4)	37 (4)	1.02 (0.61; 1.72)	0.94
Normal	377 (62)	549 (63)	1 (reference)	
Overweight/obese	172 (28)	240 (27)	1.04 (0.82; 1.32)	
Missing	30 (5)	49 (6)		
**Smoking during pregnancy**				
Non-smoker	362 (60)	668 (76)	1 (reference)	<.001
Occasional smoker	91 (15)	97 (11)	1.73 (1.27; 2.37)	
Daily smoker < 15 cigarettes	123 (20)	86 (10)	2.64 (1.95; 3.58)	
Daily smoker ≥ 15 cigarettes	23 (4)	15 (2)	2.83 (1.46; 5.49)	
Missing	6 (1)	9 (1)		
**Socio economic status**				
High level proficiency	32 (5)	80 (9)	1 (reference)	<.001
Medium level proficiency	126 (21)	269 (31)	1.17 (0.74; 1.86)	
Skilled	85 (14)	119 (14)	1.79 (1.09; 2.93)	
Student	63 (10)	95 (11)	1.66 (0.99; 2.79)	
Unskilled	135 (22)	161 (18)	2.10 (1.31; 3.35)	
Unemployed	113 (19)	78 (9)	3.62 (2.19; 5.98)	
Missing	51 (8)	73 (8)		
**Social support**				
Poor social support	90 (15)	95 (11)	1.45 (1.06; 1.98)	0.02
Good social support	480 (79)	734 (84)	1 (reference)	
Missing	35 (6)	46 (5)		
**Physically active during pregnancy**				
No	397 (66)	541 (62)	1.21 (0.97; 1.52)	0.10
Yes	182 (30)	300 (34)	1 (reference)	
Missing	26 (4)	34 (4)		
**Parity**				
0	237 (39)	400 (46)	1 (reference)	0.01
1 +	343 (57)	441 (50)	1.31 (1.06; 1.63)	
Missing	25 (4)	34 (4)		
**Supplement with multivitamin during pregnancy**				
Yes	513 (85)	745 (85)	1 (reference)	0.86
No	73 (12)	109 (12)	0.97 (0.71; 1.34)	
Missing	19 (3)	21 (2)		
**Gestation week at sample**				
<23	35 (6)	41 (5)	1.11 (0.68; 1.82)	0.61
23	103 (17)	155 (17.71)	0.87 (0.62; 1.20)	
24	153 (25)	216 (24.69)	0.92 (0.69; 1.24)	
25	152 (25)	198 (22.63)	1 (reference)	
26	80 (13)	136 (15.54)	0.77 (0.54; 1.08)	
27	30 (5)	47 (5.37)	0.83 (0.50; 1.38)	
28+	25 (4)	47 (5.37)	0.69 (0.41; 1.18)	
Missing	27 (4)	35 (4.00)		
**Season of sample**				
January-March	130 (21)	216 (24.69)	1 (reference)	0.43
April-June	152 (25)	229 (26.17)	1.10 (0.82; 1.49)	
July-September	139 (23)	189 (21.60)	1.22 (0.90; 1.66)	
October-December	158 (26)	209 (23.89)	1.26 (0.93; 1.70)	
Missing	26 (4)	32 (3.66)		

* P-value for test for homogeneity.

### Statistical methods

Using logistic regression, we estimated odds ratios (ORs) to evaluate the association between vitamin D concentrations and PPD risk. The exposure variable, vitamin D concentration, was categorized in six levels (< 15 nmol/L, 15–24 nmol/L, 25–49 nmol/L, 50–79 nmol/L, 80–99 nmol/L, ≥100 nmol/L). Vitamin D concentrations of 50–79 nmol/L were chosen as the reference category since this represents minimum sufficient levels.

The choice of six levels was based on the customary grouping of vitamin D concentrations into 4 levels (< 25 nmol/L, 25–49 nmol/L, 50–79 nmol/L and ≥80 nmol/L) [21], [25] with additional sub categorization of the lowest and highest categories to allow for investigation of extremely low and high vitamin D concentrations. However, to evaluate the association between vitamin D and PPD in the customarily used four vitamin D groups, we performed additional analyses using these four categories.

The effect of vitamin D concentrations was also modeled as a continuous variable using fractional polynomials in a logistic regression model [26] to describe the effect of vitamin D concentrations on PPD risk in more detail. Fractional polynomials offer a very flexible way of modeling continuous variables that prevents the user from having to choose arbitrary cut-points in order to categorize continuous variables; the method recognizes that it is rarely reasonable to require that the odds for adjacent categories change suddenly at the cut-point. Compared with models fitted after categorizing a continuous variable, models using fractional polynomials can yield better estimate precision using a parsimonious model for the continuous variable. Fractional polynomials also easily accommodate non-linear relationships between the variable of interest and the outcome. Statistical tests to compare different fractional polynomials are readily available. In our analyses, we chose a fractional polynomial of degree 3 since a more complex model of degree 4 did not have a significantly better fit.

In minimally adjusted analyses, we only adjusted for maternal age and year of delivery as these were the variables used in the frequency matching. In addition, we performed analyses adjusting for the potential confounders chosen a priori. Modification of the effect of vitamin D by smoking, BMI and multivitamin supplementation was evaluated by testing for multiplicative interaction by including interaction terms.

Finally, we tested whether the effect of vitamin D level on PPD risk was the same for the three highest levels of vitamin D. We tested whether our models with vitamin D in six categories could be reduced by introducing a new vitamin D variable where the three highest vitamin D levels from the previous six-level variable were collapsed into one category (≥50 nmol/L).

All significance testing was performed using likelihood ratio. Statistical analyses were carried out using Stata 12 (Stata Corporation, College Station, Texas).

## Results

The median 25(OH)D3 concentration was 55.62 nmol/L (range: 5.3–127.0 nmol/L; interquartile range [IQR]: 36.9–74.6 nmol/L) for cases and 55.60 nmol/L (range: 5.9–227.8 nmol/L; IQR: 37.5–72.4 nmol/L) for controls. Median gestational week at sampling was 24 (IQR: 24–25) for cases and 25 (IQR: 24–26) for controls. The distribution of covariates among cases and controls is presented in **Table 1**. There were significantly higher proportions of smokers and women with low socioeconomic status, poor social support and prior childbirths among cases, compared with controls.

**Table 2** presents odds ratios (ORs) for PPD by vitamin D concentration during pregnancy, using six levels of vitamin D. In the minimally adjusted model, where ORs were adjusted only for maternal age at delivery and year of delivery, there was a tendency towards a J-shaped effect of vitamin D concentration, with increased risks of PPD observed for both low (ORs of 1.70 [95% confidence interval {CI}: 0.91; 3.16], 1.05 [95% CI: 0.70; 1.58] and 1.26 [95% CI: 0.98; 1.61] for vitamin D concentrations < 15 nmol/L, 15–24 nmol/L and 25–49 nmol/L, respectively) and high (ORs of 1.30 [95% CI: 0.93; 1.82] and 1.77 [95% CI: 1.07; 2.93] for vitamin D concentrations 80–99 nmol/L and ≥100 nmol/L, respectively) concentrations of vitamin D; however, overall, the effect of vitamin D concentration was not statistically significant (p = 0.10). The estimates changed as a result of further adjustment for season, gestational week, parity, smoking, socioeconomic status, pre-pregnancy BMI, physical activity and social support and multivitamin supplementation (**Table 2**), and in the fully adjusted model, the J-shaped tendency was not as pronounced as in the minimally adjusted model (ORs of 1.35 [95% CI: 0.64; 2.85], 0.83 [95% CI: 0.50; 1.39] and 1.13 [95% CI: 0.84; 1.51] for low vitamin D concentrations, < 15 nmol/L, 15–24 nmol/L and 25–49 nmol/L, respectively, and ORs of 1.53 [95% CI: 1.04; 2.26] and 1.89 [95% CI: 1.06; 3.37] for high vitamin D concentrations, 80–99 nmol/L and ≥100 nmol/L, respectively). The overall effect of vitamin D level remained non-significant (p = 0.08) in the fully adjusted model. When only the 1,223 subjects with information on all 11 adjustment variables were included in the minimally adjusted model, the ORs changed as follows: 2.08 (95% CI: 1.05; 4.10), 0.94 (95% CI: 0.59; 1.49), 1.22 (95% CI: 0.93; 1.61), 1.48 (95% CI: 1.02; 2.14) and 1.87 (95% CI: 1.08; 3.23) for vitamin D concentrations <15, 15–24, 25–49, 80–99 and ≥100 nmol/L, respectively. The p-value for the overall effect of vitamin D level changed to p = 0.03.

**Table 2:** Odds ratio for PPD by six levels of vitamin D with adjustment for different potential confounders.

Vit. D nmol/L	Cases (n = 605)	Controls (n = 875)	Adjusted for maternal age at delivery and year of delivery 1	Additionally adjusted for season, gestation week and parity	Additionally adjusted for smoking, socio economic status, BMI, physical activity and social support	Additionally adjusted for multivitamin supplementation
	n (%)	n (%)	OR (95% CI)	OR (95% CI)	OR (95% CI)	OR (95% CI)
			n = 1480	n = 1361	n = 1253	n = 1223
< 15	22 (4)	22 (3)	1.70 (0.91; 3.16)	1.68 (0.87; 3.25)	1.30 (0.62; 2.71)	1.35 (0.64; 2.85)
15–24	46 (8)	73 (8)	1.05 (0.70; 1.58)	0.94 (0.60; 1.48)	0.83 (0.50; 1.37)	0.83 (0.50; 1.39)
25–49	203 (34)	272 (31)	1.26 (0.98; 1.61)	1.27 (0.98; 1.66)	1.14 (0.85; 1.52)	1.13 (0.84; 1.51)
50–79^1^	217 (36)	366 (42)	1 (reference)	1 (reference)	1 (reference)	1 (reference)
80–99	82 (14)	108 (12)	1.30 (0.93; 1.82)	1.40 (0.98; 1.99)	1.53 (1.05; 2.24)	1.53 (1.04; 2.26)
100+	35 (6)	34 (4)	1.77 (1.07; 2.93)	2.03 (1.19; 3.44)	1.84 (1.04; 3.26)	1.89 (1.06; 3.37)
P-value^2^			0.10	0.03	0.08	0.08

1 Matching variables.

2 P-value for test for homogeneity.

In order to allow comparison to be drawn with other studies, we also performed analyses with the four categories of vitamin D that are customarily used (<25, 25–49, 50–79 and ≥80 nmol/L). As when we used the six-level vitamin D variable, we found a J-shaped association pattern when using the variable with four levels. Thus, in the fully adjusted model ORs were 0.96 (95% CI: 0.61; 1.50), 1.13 (95% CI: 0.84; 1.51) and 1.62 (1.15; 2.30) for vitamin D levels < 25 nmol/L, 25–49 nmol/L and ≥ 80 nmol/L, respectively, compared with reference levels (50–79 nmol/L). The P-value for overall association was 0.04, substantiating the finding of higher vitamin D levels being associated with increased PPD risk. The results from the other three models were similar and are presented in a supplementary table (**Table S1**).

To examine whether the association between vitamin D concentration and PPD risk differed for different subgroups of women, we looked for effect modification by smoking, BMI and multivitamin supplementation, but none of the interactions were statistically significant (vitamin D and smoking: p = 0.31; vitamin D and BMI: p = 0.88; vitamin D and multivitamin supplementation: p = 0.22).

**Figure 1:**
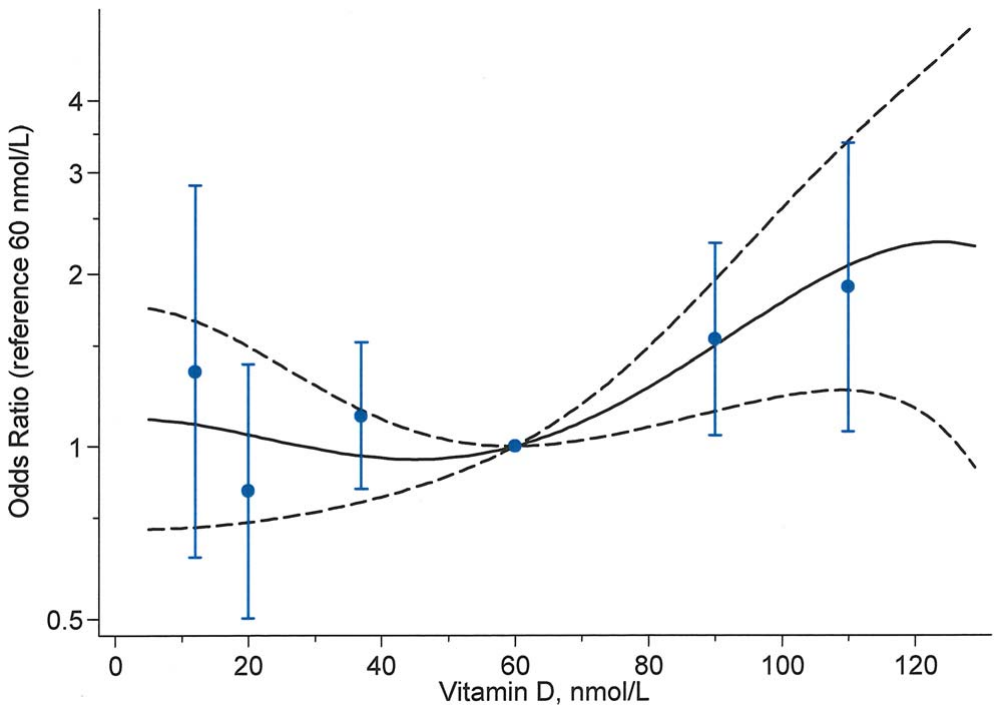
Association between postpartum depression and vitamin D. The fitted fractional polynomial model of degree 3 (FP (3)) with 95% CI in black and the categorical estimates in blue. The odds ratios are from the fully adjusted model adjusting for maternal age, year of delivery, season, gestation week, parity, smoking, socio economic status, BMI group, physical activity and social support and multivitamin supplement. The reference concentration is 60 nmol/L. The fractional polynomial part of the final model is estimated to be: log Odds =  constant+0.46·X^3^+0.27·X^3^·ln(X) -3.26·X^3^·ln(X)^2^, where X = vitamin D/100.

**Figure 1** compares odds ratios for PPD generated using a fractional polynomial with a vitamin D concentration of 60 nmol/L as the reference point (model estimates are given in the figure legend), with ORs from the fully adjusted analyses presented in **Table 2**. By using fractional polynomials, which accommodate non-linear relationships between the vitamin D variable and the outcome variable PPD, we avoided to choose cut-points to categorize the vitamin D variable. The results from the two approaches gave very similar results but use of the fractional polynomial gave a smooth curve that avoided assuming jumps in risk.

Based on the apparent higher risk of PPD in women with high vitamin D concentrations (**Table 2**), we performed an additional test comparing the women from the two highest categories of vitamin D concentrations (80–99 and ≥ 100 nmol/L) with women with normal vitamin D concentrations (50–79 nmol/L). The risks of PPD were significantly different for these three groups, regardless of the adjustment variables included (p = 0.04, p = 0.01, p = 0.02 and p = 0.02 for the four models used in Table 2, respectively), suggesting that a dose-response relationship between vitamin D concentration and PPD risk exists within normal and high vitamin D concentrations.

## Discussion

Our study showed no overall association between vitamin D status during pregnancy and PPD risk. We expected to find that low concentrations of 25(OH)D3 were associated with an increased risk of PPD, but the data provided little evidence to support this hypothesis. Unexpectedly, we found that women with higher 25(OH)D3 concentrations (> 79 nmol/L) appeared to have significantly increased risks of PPD, compared with women with concentrations in the reference category (50–79 nmol/L). The results were further supported by using fractional polynomials and an alternative categorization of the vitamin D variable.

Non-linear relationships between 25(OH)D3 and various outcomes have previously been reported. A study of neonatal vitamin D status and the risk of schizophrenia found both low and high neonatal vitamin D concentrations to be associated with increased risks of subsequent schizophrenia [7]. Similar U-shaped association patterns have been demonstrated in studies investigating the relationship between 25(OH)D3 concentrations and risk of prostate cancer in the Nordic countries [27] and active tuberculosis in Greenland [28]. Three large studies examining vitamin D status and risk of all-cause mortality [29], [30] and cardiovascular disease [31] found J-shaped, reverse J-shaped and U-shaped association patterns, respectively.

The highest concentrations of 25(OH)D3 measured in the above studies and in our study are generally not regarded as toxic [32]–[34], and consequently, investigators have put forward alternative explanations for previously observed associations between high vitamin D concentrations and adverse health outcomes. The mechanism behind the association between high concentrations of vitamin D and increased risk of prostate cancer [27] was suggested to involve 24–hydroxylase, an enzyme which is stimulated by high concentrations of plasma and intracellular 25(OH)D3 to rapidly degrade the active form of vitamin D, 1,25(OH)2D3, to the inactive 1,24,25–trihydroxyvitamin D3 metabolite. This results in low concentrations of intracellular 1,25(OH)2D3 [27], [35], which could then allow increased proliferation of malignant cells and hence an increased risk of prostate cancer. A similar 24-hydroxylase-based degradation mechanism in response to elevated vitamin D concentrations may apply in PPD. Low concentrations of 1,25(OH)2D, whether resulting directly from low 25(OH)D3 concentrations or following degradation in response to abnormally high 25(OH)D3 concentrations, could then be speculated to provide insufficient stimulation for vitamin D receptors in the brain, resulting in improper functioning of vitamin D-dependent hormonal processes in the brain that otherwise would prevent mood disorders. Such improper functioning would then lead to the observation of increased PPD risk in women with both very high and very low serum vitamin D concentrations.

Alternatively, genetic variation may play a role. It has been hypothesized that single-nucleotide polymorphisms in certain population subgroups may impair the conversion of 25(OH)D3 to the active 1,25(OH)2D3 [7], resulting in relatively high circulating serum concentrations of 25(OH)D3 but lower than optimal concentrations of 1,25(OH)2D3. Thus, high concentrations of 25(OH)D3 may not lead to high 1,25(OH)2D3 concentrations in these individuals but rather to low concentrations, such that the relationship between high measured vitamin D concentrations and PPD risk would be similar in strength to that in individuals with low 25(OH)D3 concentrations, resulting in a U-shaped relationship.

Although not evaluated in our study, vitamin D binding protein (group-specific component, GC) concentrations may also have affected our findings, since GC plays a key role in the bioavailability of 25(OH)D3 and the active form 1,25(OH)2D3 [36]. The assay used for estimating vitamin D status measures total 25(OH)D3, i.e. the sum of protein-bound and free 25(OH)D3. It is generally believed that only free 25(OH)D3 is biologically active and able to enter cells and bind to the vitamin D receptor [37]. It is well known that GC concentrations increase during pregnancy [38], and the higher the concentration of GC protein, the lower the proportion of total 25(OH)D3 that is free [36]. Women with higher GC concentrations may have reduced metabolic outflow of 25(OH)D3 and therefore less 25(OH)D3 will be available for conversion to the active 1,25(OH)2D3, lengthening the functional half-life of 25OHD3 [37]. Consequently, women with higher than normal GC concentrations could have higher total 25OHD3, and when tested with routine assays for 25(OH)D3, as we did in our study, women with high GC will have high-normal total 25(OH)D3, but lower than expected free 25(OH)D3. Thus, a high measured concentration of 25(OH)D3 may conceal a low free fraction of 25(OH)D3, and consequently a low concentration of the biological active 1,25(OH)2D3. This could produce a U- or J-shaped relationship between 25(OH)D3 concentrations and PPD risk. Testing the above scenario by measuring the concentration of GC in maternal serum and deriving an estimate of free 25(OH)D3, as is routinely done in the assessment of free testosterone, would be a logical extension of our study. Estimation of free 25(OH)D3 would be useful in studies attempting to evaluate associations between vitamin D status and various health outcomes.

If the mechanism underlying our finding of an association between high 25(OH)D3 concentrations and PPD risk is actually related to low concentrations of active vitamin D, as a result either of degradation and thereby low intracellular 1,25(OH)2D3 or limited conversion of 25(OH)D3 to the active 1,25 (OH)2D3 due to genetics or due to a reduced free fraction of 25(OH)D3, this would suggest that low concentrations of active vitamin D increase PPD risk.

Most of the women in our study had 25(OH)D3 concentrations between 50 and 79 nmol/L, which is characterised as sufficient by the Danish National Board of Health. Women with concentrations between 25 and 49 nmol/L, characterised as insufficient, also constituted a large group, substantiating a previous report of a high prevalence of vitamin D insufficiency among pregnant women [20]. Although samples were stored for 9–15 years prior to measurement of 25(OH)D3, the 25(OH)D3 concentrations reported are likely indicative of the concentrations at the time of sampling, since 25(OH)D3 appears to be stable in serum over long storage periods [39]. If some degradation did occur, matching on year of delivery should have minimized the risk of differential misclassification by unequal degradation among samples.

Our study was nested in a large prospective cohort, the DNBC, which recruited approximately 35% of pregnant women in Denmark between 1996 and 2002 [24]. The proportion of PPD cases in the DNBC corresponds well with the proportion of PPD cases identified in a recent register based population-wide cohort study using a similar definition (unpublished data). Although the participants in the DNBC were generally more health-conscious and better-off than non-participants [40] such selection would result in biased estimates only if the likelihood of being followed in the cohort depended on both the exposure and the outcome, which in other settings in DNBC has been shown not to be the case [41]. As in any observational study we cannot exclude the possibility that our findings are affected by confounders not adjusted for. However, we adjusted for many confounders selected a priori based on previous literature in the field, making it difficult to identify an unmeasured confounder that can explain our findings.

By directly measuring vitamin D concentrations in biological samples, rather than estimating them based on reported food intake, we greatly reduced the possibility of misclassification of vitamin D concentrations. With regards to the identification of PPD cases, we relied on high-quality population register data with close to complete follow-up and used a case definition developed for previous studies [42]. Whether our use of register data on antidepressant use as a measure of PPD captures the women most relevant to a study of PPD is subject to discussion, since these women may not necessarily fulfill the DSM-IV diagnostic criteria for PPD. However, in Denmark such medication can only be prescribed by a physician, regulation and surveillance of the use of medication is quite strict, and the sensitivity of antidepressant use as a proxy for major depression has been evaluated by Thielen et al. [43] to be 50%. Most importantly, the specificity was >90% [43], making information bias in this case-control study with a rare outcome unlikely.

The public health implications of our findings are ambiguous as long as the underlying mechanism is unclear. The highest vitamin D concentrations in our study were far below the concentrations which are generally perceived as toxic, and we find it unlikely that the association between these higher vitamin D concentrations and increased risk of PPD reflects a direct harmful effect of vitamin D. However, the study indicates that complex biological mechanisms are involved in the relationship between vitamin D during pregnancy and the risk of PPD, suggesting that recommendations of vitamin D supplementation to pregnant women should be considered with caution.

## Supporting Information

Table S1 Odds ratio for PPD by four levels of vitamin D with adjustment for different potential confounders.
(DOCX)
