# Supplementary material for: Vitamin D Status during Pregnancy and the Risk of Subsequent Postpartum Depression: A Case-Control Study
Source: PLoS One. 2013 Nov 27;8(11):e80686. doi: 10.1371/journal.pone.0080686 (PMC3842313; doi:10.1371/journal.pone.0080686)
Supplement: Table S1 — Odds ratio for PPD by four levels of vitamin D with adjustment for different potential confounders. (DOCX) [file pone.0080686.s001.docx]

Table S1. Odds ratio for PPD by four levels of vitamin D with adjustment for different potential confounders.

| Vit. D nmol/L | Cases (n=605) | Controls (n=875) | Adjusted for maternal age at delivery and year of delivery ^1^ | Additionally adjusted for season, gestation week and parity | Additionally adjusted for smoking, socio economic status, BMI, physical activity and social support | Additionally adjusted for multivitamin supplementation |
| --- | --- | --- | --- | --- | --- | --- |
|  | n (%) | n (%) | OR (95% CI) | OR (95% CI) | OR (95% CI) | OR (95% CI) |
|  |  |  | n = 1480 | n = 1361 | n = 1253 | n = 1223 |
| < 25 | 68 (11) | 95 (11) | 1.20 (0.84; 1.71) | 1.12 (0.75; 1.66) | 0.94 (0.61; 1.46) | 0.96 (0.61; 1.50) |
| 25-49 | 203 (34) | 272 (31) | 1.26 (0.98; 1.61) | 1.27 (0.97; 1.66) | 1.14 (0.85; 1.52) | 1.13 (0.84; 1.51) |
| 50-79^1^ | 217 (36) | 366 (42) | 1 (reference) | 1 (reference) | 1 (reference) | 1 (reference) |
| 80+ | 117 (19) | 142 (16) | 1.41 (1.05; 1.90) | 1.54 (1.13; 2.12) | 1.61 (1.15; 2.27) | 1.62 (1.15; 2.30) |
| P-value^2^ |  |  | 0.10 | 0.04 | 0.04 | 0.04 |

^1^Matching variables.

^2^P-value for test for homogeneity.
